# Activity of Rezafungin Against Echinocandin Non–wild type *Candida glabrata* Clinical Isolates From a Global Surveillance Program

**DOI:** 10.1093/ofid/ofae702

**Published:** 2025-03-03

**Authors:** Mariana Castanheira, Lalitagauri M Deshpande, John H Kimbrough, Marisa Winkler

**Affiliations:** Element Iowa City (JMI Laboratories), North Liberty, Iowa USA; Element Iowa City (JMI Laboratories), North Liberty, Iowa USA; Element Iowa City (JMI Laboratories), North Liberty, Iowa USA; Element Iowa City (JMI Laboratories), North Liberty, Iowa USA

**Keywords:** breakpoints, *Candida glabrata*, echinocandins, FKS mutations, rezafungin

## Abstract

Among 1463 *Candida glabrata* isolates collected in 39 US hospitals, 91 (6.2%) were non–wild type to ≥1 echinocandins (ECH-NWT) when tested by the Clinical and Laboratory Standards Institute (CLSI) reference broth microdilution method and interpretative criteria. Rezafungin breakpoints established by the US Food and Drug Administration (FDA) were also applied. ECH-NWT isolates were noted in all US census divisions, and 71 (79.0% of ECH-NWT) carried FKS hot spot (HS) alterations. S663P in FKS2 HS1 (31 isolates) was the most common alteration, followed by substitutions/deletions in position F659 in FKS2 HS1 (14 isolates) and S629P in FKS1 HS1 (9 isolates). Six isolates had substitutions in the HSs of FKS1 and FKS2, and 8 other alterations were noted in the 11 remaining isolates. When CLSI/FDA breakpoints were applied, rezafungin was active against 97.5%/95.3% and 59.3%/23.9% of the overall *C glabrata* and ECH-NWT isolates, respectively. Anidulafungin, caspofungin, and micafungin inhibited 93.9%/13.2%, 95.7%/33.0%, and 95.6%/29.7% of the overall *C glabrata*/ECH-NWT isolates. Isolates that did not harbor FKS HS substitutions were more susceptible to echinocandins when compared with isolates with substitutions (47.4%–100% and 4.2%–49.3%; lowest for anidulafungin and highest for rezafungin per the CLSI breakpoint). Isolates harboring the FKS2 HS1 S663P alterations were more resistant to echinocandins—3.2% susceptible (anidulafungin) to 35.5% (rezafungin CLSI breakpoint)—when compared with other single alterations. Rezafungin dosing and pharmacokinetic/pharmacodynamic characteristics allow for coverage of higher minimum inhibitory concentration values, making this agent an attractive option for some isolates that carry FKS alterations and still demonstrate rezafungin-susceptible minimum inhibitory concentration values.

## Teaser

We evaluated the echinocandin susceptibility of echinocandin non–wild type *Candida glabrata* isolates, including FKS mutants. By applying the breakpoints of the Clinical and Laboratory Standards Institute, rezafungin was more active than other echinocandins, but the activity of in-class agents was similar when the US Food and Drug Administration breakpoint was applied.

*Candida glabrata*, recently renamed *Nakaseomyces glabratus*, is the second- or third-most common yeast species causing invasive candidiasis, depending on the geographic region and patient population [1–3]. In an evaluation of the first 10 years of the SENTRY Antifungal Surveillance Program, Pfaller et al identified *C glabrata* as the second-most common *Candida* species after *C albicans* in North America, Europe, and Asia-Pacific and the third-most common *Candida* species in Latin America following *C albicans* and *C parapsilosis* [4].

Unlike most species that were previously identified as part of the genus *Candida*, *C glabrata* is a haploid organism [1]. Mutations in the single allele of genes involved in antifungal resistance can confer elevated minimum inhibitory concentration (MIC) values for these agents in this species [5]. Fluconazole resistance in *C glabrata* clinical isolates is common and varies by geographic region. Surveillance data from 2018 to 2019 reported that overall fluconazole resistance in *C glabrata* was 6.1%, but rates were greater in North America (8.1%) and Europe (5.9%) when compared with Asia-Pacific (2.7%) and Latin America, where resistant isolates were not observed [6].

Due to the emergence of azole resistance in *Candida* species, including *C glabrata*, and the safety profile of the echinocandins, the Infectious Diseases Society of America recommends an echinocandin as a first-line therapy for candidemia and other invasive candida infections [7]. Echinocandin resistance in *Candida* species is still uncommon when compared with azole resistance, but *C glabrata* has the highest resistance rates to these agents. In an analysis of the SENTRY Antifungal Surveillance Program [4] from 2006 to 2016, echinocandin resistance was <0.1% for *C albicans*, *C tropicalis*, and *C parapsilosis*, but 1.7%, 2.2%, and 3.5% of the *C glabrata* isolates were resistant to micafungin, anidulafungin, and caspofungin, respectively. More recent data from the same global surveillance program demonstrated that 2.4%, 1.7%, and 2.1% of the 289 *C glabrata* isolates collected in 2019 to 2020 were resistant to anidulafungin, caspofungin, and micafungin [8]. However, these rates can be much higher in some locations. In a landmark study published by Alexander et al [9] surveying 10 years of *C glabrata* isolates causing candidemia in a university-based hospital in the United States, resistance to an echinocandin increased from 4.9% to 12.3% in the study period. In addition, fluconazole resistance was as high as 30% by the end of the study period, and 14% of the fluconazole-resistant isolates were also resistant to an echinocandin.

Rezafungin is a long-acting echinocandin that was approved in 2022 for the treatment of candidemia and invasive candidiasis in patients aged ≥18 years who have limited or no alternative treatment options [10]. Breakpoints for rezafungin against 5 *Candida* species were approved by the Food and Drug Administration (FDA; susceptible at ≤0.12 mg/L) [11], and slightly different breakpoints were recently approved by Clinical and Laboratory Standards Institute (CLSI; susceptible at ≤0.5 mg/L). The CLSI susceptibility breakpoint for rezafungin against *C glabrata* is 2 dilutions higher [12] than the breakpoints adopted by the FDA due to the pharmacokinetic/pharmacodynamic (PK/PD) profile of rezafungin, which allows for higher concentrations of this agent in the bloodstream and at other sites of infection.

In this study, we evaluated the epidemiology of echinocandin non–wild type *C glabrata* isolates collected in US hospitals from 2014 to 2022 as part of the SENTRY Antifungal Surveillance Program [4]. We tested the activity of all clinically available echinocandins against this collection using the FDA and CLSI breakpoint criteria. All echinocandin non–wild type *C glabrata* isolates had the genes encoding FKS sequenced, and the results were analyzed with the susceptibility profile of the isolates carrying FKS alterations.

## MATERIALS AND METHODS

### Fungal Isolates

A total of 1463 *C glabrata* clinical isolates were collected from invasive candidal infections from 2014 to 2022 in 39 US hospitals. Participating institutions were requested to send up to 40 consecutive fungal isolates collected from sterile sites. All isolates were shipped to a central location (Element Materials/JMI Laboratories) and subcultured on CHROMagar Candida (Sigma-Aldrich) to confirm purity. Organism identification was confirmed by matrix-assisted laser desorption ionization–time-of-flight mass spectrometry or molecular methods as previously described [13].

### Antifungal Susceptibility Testing

*C glabrata* isolates were susceptibility tested by the reference broth microdilution method described by the CLSI [14] against anidulafungin, caspofungin, micafungin, rezafungin, and fluconazole. Quality control was monitored by concomitantly testing *C parapsilosis* ATCC 22019 and *C krusei* ATCC 6258, and results were interpreted according to M27 and M27M44S guidelines [14, 15]. CLSI breakpoints and epidemiologic cutoff values were applied [15, 16]. Additionally, the FDA-adopted susceptible breakpoint (≤0.12 mg/L) and the recently confirmed CLSI susceptible breakpoint (≤0.5 mg/L) were applied for rezafungin.

### Whole Genome Sequencing and Analysis

Non–wild type isolates to anidulafungin (MIC >0.25 mg/L), micafungin (MIC >0.03 mg/L), or rezafungin (MIC >0.12 mg/L) [15] were submitted to analysis of the FKS genes by whole genome sequencing [6, 17]. Since epidemiologic cutoff values for the CLSI method are not available for caspofungin against *C glabrata* due to the variability in caspofungin testing, we did not use that agent to qualify isolates for this study. However, CLSI clinical breakpoints available for this agent were applied for susceptibility pattern analysis [15]. Total genomic DNA was used as input material for library construction prepared with the Nextera XT library construction protocol and index kit (Illumina), following the manufacturer's instructions. Sequencing was performed on a MiSeq Sequencer (Illumina). Reads were error corrected by BayesHammer, and each sample was assembled with a reference-guided assembly in DNASTAR SeqMan NGen version 14.0. FKS sequences were compared with those of the echinocandin-susceptible *C glabrata* ATCC 90030.

## RESULTS

### Epidemiology and Distribution of the Echinocandin Non–wild type *C glabrata* Isolates

Among 1463 of *C glabrata* isolates, 91 (6.2%) were non–wild type to at least 1 echinocandin: 85 (5.8%) to micafungin, 69 (4.7%) to rezafungin, and 55 (3.8%) to anidulafungin. Echinocandin non–wild type *C glabrata* isolates were recovered in 19 US states and all US census divisions. The Middle Atlantic (20 isolates), East North Central (15), Pacific (14), and Mountain (13) divisions had the most isolates, while the West North Central (2 isolates) and New England (3) divisions had the fewest numbers of echinocandin non–wild type *C glabrata* isolates.

FKS hot spot (HS) alterations were noted among 71 (79.0%) of the echinocandin non–wild type *C glabrata* isolates available for analysis (Table 1). One isolate could not be recovered for sequencing. FKS2 HS1 was the most common site of alterations, as observed in 55 isolates, followed by alterations in FKS1 HS1, as harbored by 15 isolates. Six isolates harbored alterations in both HSs, and no isolates had changes in FKS1 HS2 or FKS2 HS2.

**Table 1.** Genetic and Phenotypic Characteristics of Echinocandin Non–wild type *Candida glabrata* Isolates From US hospitals

			MIC Range, mg/L					
FKS Status	No. of Isolates	State (No. of Isolates If >1)	Rezafungin	Anidulafungin	Caspofungin	Micafungin	No. of Isolates per WGS	Non-HS Mutations (No. of Isolates If >1)
**FKS1 HS1**								
F625S	3	CA, GA, VA	0.25–2	0.5–2	0.12–2	0.06–0.5	1	
L628R	1	NJ	0.12	0.12	0.03	0.06		
S629P	9	CA, CO, IN, MA, NJ, NY (3), WA	0.12–2	0.25–4	0.12–>4	0.06–4	8	FKS1 G14S FKS2 G1284R FKS1 F1335L + FKS2 W1495* FKS2 Q1704*
L630Q	1	WA	0.25	0.25	0.12	0.06		
D632E	1	MI	0.5	1	1	0.25	1	FKS1 D892E + FKS2 Q388*
**FKS2 HS1**								
F659 deletion(9)/S(2)/Y(2)/L(1)	14	AL, CA (2), CO (3), MI, NJ, NY (4), VA (2)	0.06–4	0.06–4	0.06–>4	0.03–4	9	FKS1 G14S (3)
L662W	1	MI	0.25	0.25	0.5	0.06		
S663P	31	AL, CA, CO, IA, KS, KY (6), MA, MI (2), NJ (2), NY (5), OH (3), TX (2), UT, WA (4)	0.06–2	0.12–4	0.06–>4	0.06–4	25	FKS1 G14S FKS1 G14S, Y477H, F1727C + FKS2 T926P FKS1 G14S, A1436V FKS1 G14S + FKS2 T926P (7) FKS2 F30V (2) FKS2 N120K FKS2 A122V
R665G	2	LA, CO	0.12–0.25	0.5	0.25	0.25	2	
D666Y	1	VA	0.25	0.25	0.12	0.03		
P667H	1	CO	0.12	0.25	0.25	0.12	1	FKS1 G14S
**Double mutations**								
F625S + F659	1	IN	1	2	2	0.5		
S629P + S663P	2	TX, OH	2–4	4	>4	4	2	FKS1 T307-F1055 deletion + FKS2 P1150Q, T1155S, K1164N
S629P + R665G	1	WA	0.5	1	0.5	1	1	FKS2 S481P
R635G + I661F	1	UT	0.12	0.25	0.25	0.06	1	FKS1 A1633T
Non-HS insertion + S663P	1	VA	2	4	4	4	1	FKS1 A597 insertion
**No HS mutation**	19	CA, CO (4), IN (2), LA, NY (2), OH (2), TX (3), VA (2), VT, WA	0.015–0.12	0.03–0.5	0.03–0.25	0.015–2	13	FKS1 G14S + FKS2 T926P (3) FKS1 G14S + FKS2 T926P, K1357E FKS1 I634V, K1323E FKS1 E1047G FKS2 F30V FKS2 F384Y FKS2 W715L FKS2 K1357E

Abbreviations: HS, hot spot; MIC, minimum inhibitory concentration; WGS, whole genome sequencing.

The most common alteration was the FKS2 HS1 S663P, which was observed alone in 31 isolates (34.1% of the echinocandin non–wild type, 43.7% of all FKS alterations). Isolates harboring this change were recovered in 14 US states. Fourteen isolates carried changes in position 659 of the FKS2 HS1: 9 harbored a deletion of the phenylalanine and the remaining 5 had amino acid substitutions in this position (F→Y [2], F→S [2], or F→L [1]). The alteration FKS1 HS1 S629P was noted in 9 isolates collected in 7 states. Eleven remaining isolates carried 8 other single alterations, either in FKS1 HS1 (4 alterations, 6 isolates) or FKS2 HS1 (4 alterations, 5 isolates). A total of 6 isolates carried double alterations in FKS1 and FKS2 HS1: F625 plus F659 (1 isolate), S629P plus S663P (2), S629P plus R665G (1), R635G plus I661F (1), or an insertion of 1 amino acid at position 597 of FKS1 that shifted the HS in addition to S663P (1).

In addition to the HS alterations, various isolates that were submitted to whole genome sequencing (65/91, 71.4%) and had the entire FKS sequence available harbored FKS non-HS alterations that included the early termination of the gene and large deletions (Table 1). Notably, non-HS alterations were observed in 77.0% (10/13) of the sequenced isolates that did not carry FKS alterations. These numbers were significantly lower among isolates harboring FKS mutations (27/52, 51.9%, *P* < .01; odds ratio, 3.086 [95% CI 0.769–12.519]).

A total of 20 echinocandin non–wild type *C glabrata* isolates displayed rezafungin MIC values at 0.25 mg/L, which would be considered susceptible by applying CLSI breakpoints and resistant by applying the FDA breakpoints (data not shown). These isolates displayed anidulafungin MIC values ranging from 0.25 to 0.5 mg/L, caspofungin from 0.06 to 0.5 mg/L, and micafungin from 0.15 to 0.25 mg/L. Six of these isolates carried no FKS HS alterations. Among the 14 isolates displaying FKS HS alterations, the substitutions/deletions noted were FKS1 HS1 S629P (2 isolates), F625S (1), and L630Q (1) and FKS2 HS1 deletion of F659 (2), F659Y (2), S663P (2), D666Y (1), F625S (1), F659L (1), L630Q (1), L662W (1), and R665G (1).

### Susceptibility Profile of the Echinocandin Non–wild type *C glabrata* Isolates

**Figure 1.**
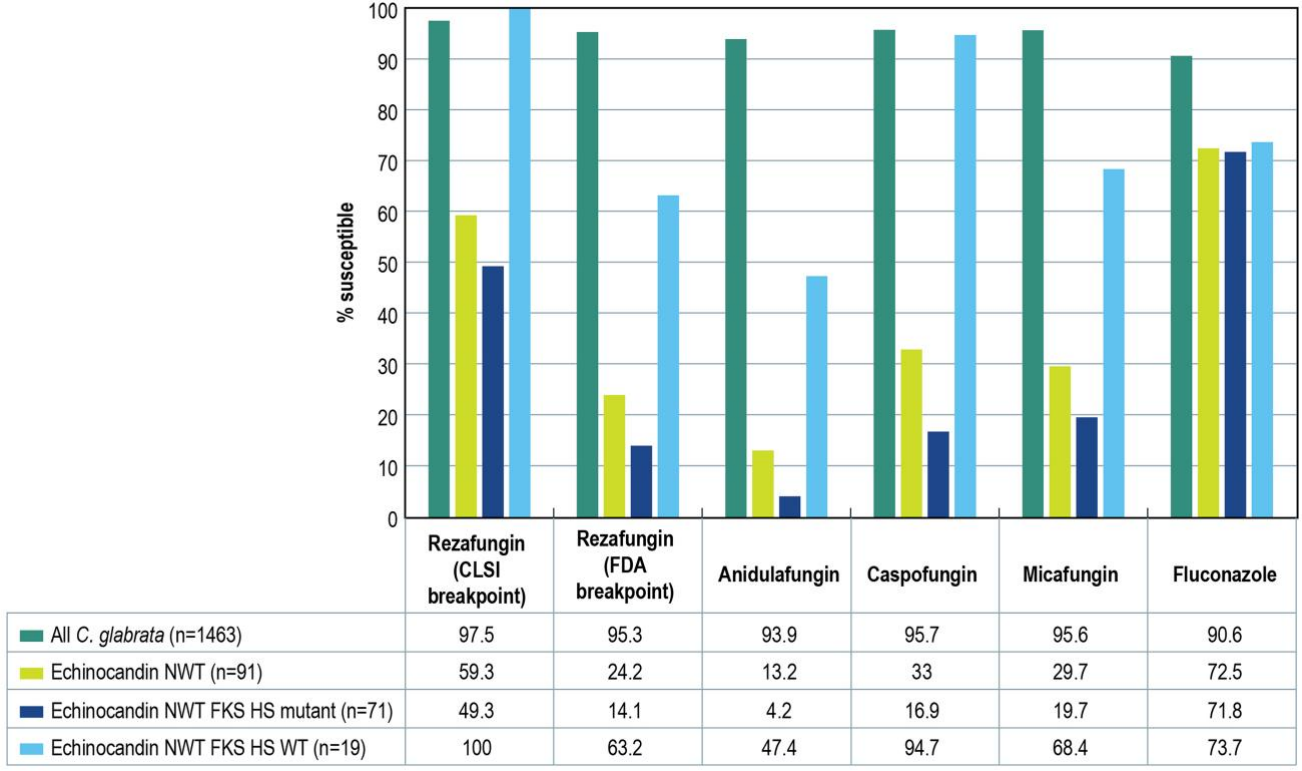
Susceptibility patterns of *Candida glabrata* collected in 39 US hospitals. One echinocandin NWT isolate was not available for sequencing and could not be categorized for FKS alterations. CLSI, Clinical and Laboratory Standards Institute; FDA, Food and Drug Administration; HS, hot spot; NWT, non–wild type; WT, wild type.

As expected, echinocandin non–wild type *C glabrata* isolates displayed lower echinocandin susceptibility rates when compared with the overall *C glabrata* collection (Table 2, Figure 1). Rezafungin inhibited 97.5% of the overall *C glabrata* isolates and 59.3% of the echinocandin non–wild type isolates when the CLSI breakpoint was applied. These rates were 95.3% and 23.9%, respectively, when based on FDA breakpoints. Anidulafungin, caspofungin, and micafungin inhibited 13.2%, 33.0%, and 29.7% of the echinocandin non–wild type isolates and 93.9%, 95.7%, and 95.6% of the overall *C glabrata* set. When isolates were stratified by FKS HS alteration status, rezafungin inhibited 49.3% of the echinocandin non–wild type isolates that harbored FKS HS mutations and 100.0% of the isolates that did not carry these alterations when the CLSI breakpoints were applied, but these susceptibility rates were 14.1% and 63.2% when the FDA breakpoints were applied. Anidulafungin, caspofungin, and micafungin inhibited 4.2%, 16.9%, and 19.7% of the isolates carrying FKS HS alterations and 47.4%, 94.7%, and 68.4% of the isolates that did not carry such alterations.

**Table 2.** Activity of Echinocandins Tested Against *Candida glabrata* Isolates Collected in 39 US hospitals

	MIC, mg/L											MIC, %	
Organism: Antifungal Agent	0.008	0.015	0.03	0.06	0.12	0.25	0.5	1	2	4	>	50%	90%
**All *C glabrata* (n = 1463)**													
Rezafungin, mg/L												0.03	0.12
No.	53	142	551	530	118	20	12	19	16	2	…		
Cumulative %	3.6	13.3	51	87.2	95.3	96.7	97.5	98.8	99.9	100	…		
Anidulafungin, mg/L												0.06	0.12
No.	13	64	228	715	354	34	7	18	20	10	…		
Cumulative %	0.9	5.3	20.8	69.7	93.9	96.2	96.7	97.9	99.3	100	…		
Caspofungin, mg/L												0.03	0.06
No.	8	213	786	332	61	17	12	13	6	6	9		
Cumulative %	0.5	15.1	68.8	91.5	95.7	96.9	97.7	98.6	99	99.4	100		
Micafungin, mg/L												0.015	0.03
No.	269	900	209	21	8	17	11	14	8	6	…		
Cumulative %	18.4	79.9	94.2	95.6	96.2	97.3	98.1	99	99.6	100	…		
**Echinocandin NWT (n = 91)**													
Rezafungin, mg/L												0.5	2
No.	…	1	2	2	17	20	12	19	16	2	…		
Cumulative %	…	1.1	3.3	5.5	24.2	46.2	59.3	80.2	97.8	100	…		
Anidulafungin, mg/L												1	4
No.	…	…	1	3	8	24	7	18	20	10	…		
Cumulative %	…	…	1.1	4.4	13.2	39.6	47.3	67	89	100	…		
Caspofungin, mg/L												0.25	4
No.	…	…	2	13	15	16	11	13	6	6	9		
Cumulative %	…	…	2.2	16.5	33	50.5	62.6	76.9	83.5	90.1	100		
Micafungin, mg/L												0.25	2
No.	…	1	5	21	8	17	11	14	8	6	…		
Cumulative %	…	1.1	6.6	29.7	38.5	57.1	69.2	84.6	93.4	100	…		
**Echinocandin NWT FKS HS mutant (n = 71)**													
Rezafungin, mg/L												1	2
No.	…	…	0	2	8	14	11	19	15	2	…		
Cumulative %	…	…	0	2.8	14.1	33.8	49.3	76.1	97.2	100	…		
Anidulafungin, mg/L												1	4
No.	…	…	0	1	2	15	6	18	20	9	…		
Cumulative %	…	…	0	1.4	4.2	25.4	33.8	59.2	87.3	100	…		
Caspofungin, mg/L												0.5	>4
No.	…	0	1	5	6	15	11	13	6	6	8		
Cumulative %	…	0	1.4	8.5	16.9	38	53.5	71.8	80.3	88.7	100		
Micafungin, mg/L												0.5	2
No.	…	0	2	12	6	15	10	14	6	6	…		
Cumulative %	…	0	2.8	19.7	28.2	49.3	63.4	83.1	91.5	100	…		
**Echinocandin NWT FKS WT (n = 19)**													
Rezafungin, mg/L												0.12	0.25
No.	0	1	2	0	9	6	1	…	…	…	…		
Cumulative %	0	5.3	15.8	15.8	63.2	94.7	100	…	…	…	…		
Anidulafungin, mg/L												0.25	0.25
No.	…	0	1	2	6	9	1	…	…	…	…		
Cumulative %	…	0	5.3	15.8	47.4	94.7	100	…	…	…	…		
Caspofungin, mg/L												0.12	0.12
No.	…	0	1	8	9	1	…	…	…	…	…		
Cumulative %	…	0	5.3	47.4	94.7	100	…	…	…	…	…		
Micafungin, mg/L												0.06	0.5
No.	0	1	3	9	2	2	1	0	1	…	…		
Cumulative %	0	5.3	21.1	68.4	78.9	89.5	94.7	94.7	100	…	…		

One echinocandin NWT isolate was not available for sequencing and could not be categorized for FKS alterations.

Abbreviations: HS, hot spot; MIC, minimum inhibitory concentration; NWT, non–wild type; WT, wild type.

Fluconazole was active against 90.6% of the 1463 *C glabrata* isolates, 72.5% of the echinocandin non–wild type isolates, 71.8% of the echinocandin non–wild type isolates carrying FKS HS mutations, and 73.7% of the isolates carrying no FKS changes.

**Figure 2.**
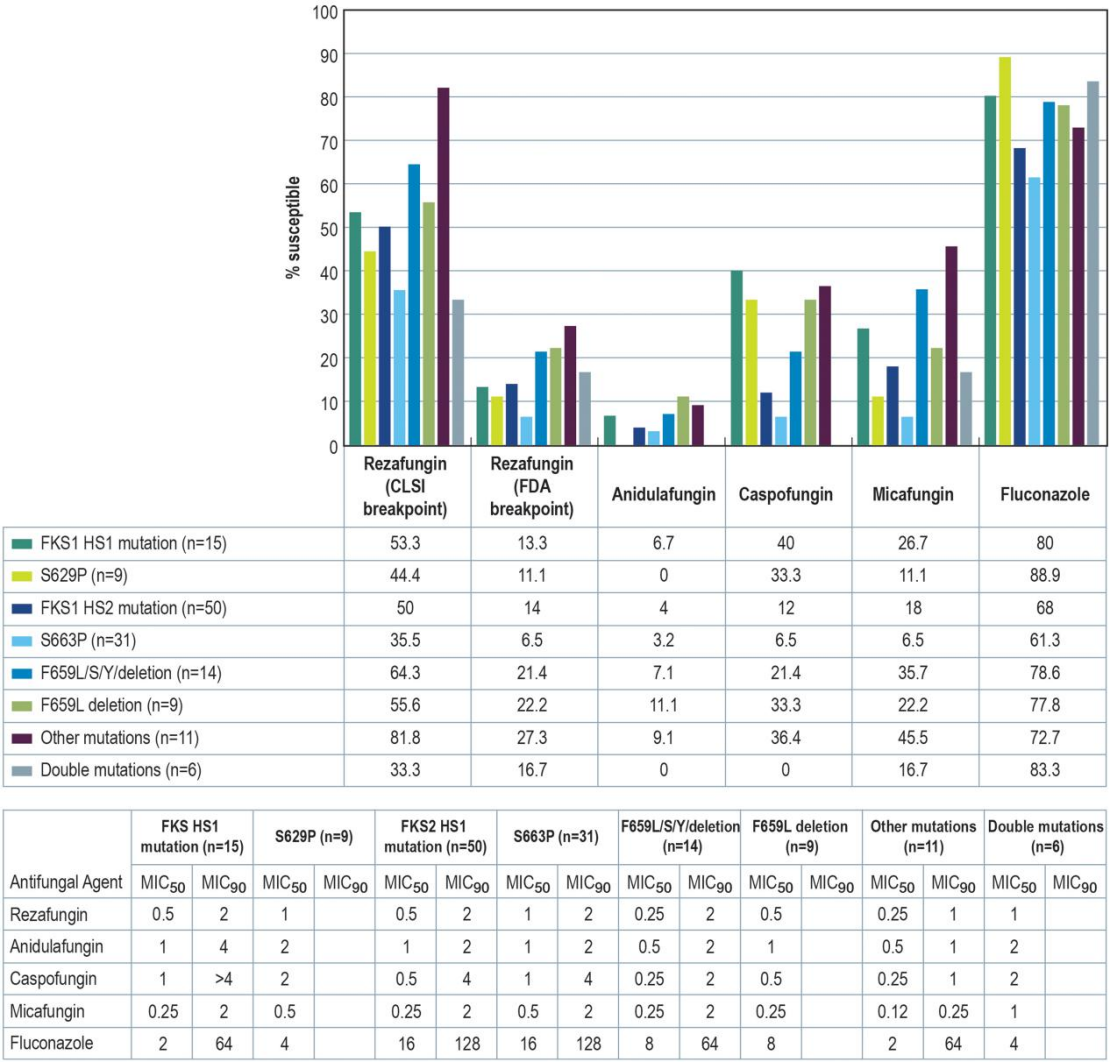
Susceptibility patterns of *Candida glabrata* collected in 39 US hospitals stratified by genotype. CLSI, Clinical and Laboratory Standards Institute; FDA, Food and Drug Administration; MIC, minimum inhibitory concentration.

Isolates harboring different FKS mutations exhibited alternative susceptibility profiles against echinocandin agents (Figure 2). Rezafungin was active against either 53.3% or 13.3% of the 15 isolates harboring FKS1 HS1 alterations when the CLSI or FDA breakpoints were applied, respectively. Anidulafungin, caspofungin, and micafungin were active against 6.7%, 40.0%, and 26.7% of these isolates. These values were slightly lower when only 9 isolates harboring the FKS1 HS1 S629P were analyzed. Rezafungin, anidulafungin, caspofungin, and micafungin were active against 44.4%/11.1% (CLSI/FDA breakpoint), 0.0%, 33.3% and 11.1% of these isolates.

The susceptibility rate for 50 isolates carrying FKS2 HS1 alterations was lower for the other echinocandins but higher for rezafungin when compared with other FKS alteration groups. Rezafungin inhibited 50.0%/14.0% (CLSI/FDA breakpoint) of these isolates while anidulafungin, caspofungin, and micafungin inhibited 4.0%, 12.0%, and 18.0%, respectively. The 31 isolates carrying FKS2 HS1 S663P were considerably more resistant to all echinocandins: rezafungin was active against 35.5%/6.5% (CLSI/FDA), anidulafungin was active against 3.2%, and caspofungin and micafungin inhibited 6.5% of the isolates.

The activity of the echinocandins was much higher among 11 isolates carrying less common mutations in FKS1 HS1 or FKS2 HS1. Rezafungin was active against 81.8% or 27.3% of these isolates with application of the CLSI or FDA breakpoint, respectively. Anidulafungin, caspofungin, and micafungin were active against 9.1%, 36.4%, and 45.5% of these isolates. Conversely, isolates harboring mutations in both FKS HS regions were more resistant to these agents: 33.3%/16.7% (CLSI/FDA), 0%, 0%, and 16.7% of these were susceptible to rezafungin, anidulafungin, caspofungin, and micafungin.

Fluconazole susceptibility rates ranged from 61.3% to 88.9% and were the lowest against isolates harboring a FKS2 HS1 S663P alteration and highest against those carrying FKS1 HS1 S629P.

## DISCUSSION

*C glabrata* is the most common non-*albicans Candida* species causing invasive infections in various geographic regions and clinical settings [1, 4, 18]. The Centers for Disease Control and Prevention highlighted *C glabrata* as one of the contributors to increasing rates of drug resistance among *Candida* species [19]. Due to the elevated mortality rates of candidemia and invasive candidiasis and an increase in fluconazole resistance rates in various *Candida* species, an echinocandin is preferred for the treatment of these infections [7]. Yet, among organisms previously belonging to the genus *Candida*, *C glabrata* displays the highest rates of echinocandin resistance [4, 20, 21]. In this 9-year surveillance of 39 US hospitals, 6.2% of the isolates were non–wild type to at least 1 echinocandin. Notably, echinocandin non–wild type isolates were observed in all US census divisions, highlighting the importance of understanding epidemiology and the ability of laboratories to identify these isolates. These numbers are still low when compared with resistance against certain antibacterial agents. However, any resistance among fungal pathogens might leave fewer treatment options for patients when we take the following into consideration: first, there are fewer classes of antifungal agents; second, resistance mechanisms that inhibit glucan synthase confer cross-resistance to echinocandins and the triterpenoid ibrexafungerp [22–24]; third, resistance mechanisms that confer cross-resistance to azoles and amphotericin B act in the ergosterol biosynthesis pathway [25].

Resistance to the echinocandins is usually caused by alterations in the target of these agents: the 1,3-β-D-glucan synthase subunits. While in most *Candida* species alterations in FKS1 causes resistance to echinocandins, in *C glabrata* resistance can be caused by changes in FKS1 or its homologue, FKS2 [5, 20]. Among *C glabrata* clinical isolates, FKS2 mutations seem to be more prevalent: the alteration S663P is the most commonly reported in this and various other studies [9, 26, 27]. The alteration S663P was followed in number of isolates by substitutions or deletions in F659 in FKS2 HS1 (14 isolates) and by the FKS1 HS1 substitution S629P (9 isolates). Of note, 6 isolates carried mutations in the HS regions of both FKS genes.

Very few studies have enough FKS mutant isolates to allow for stratification of the isolates by FKS mutation or mutation type for analysis of their susceptibility patterns, but this analysis could aid an understanding on how mutations affect the susceptibility of various echinocandins among clinical isolates with similar alterations. We observed that when compared with isolates carrying FKS1 HS substitutions, FKS2 HS alterations cause higher resistance rates to all echinocandins and to fluconazole as well. Additionally, among the FKS2 HS substitutions, isolates harboring S663P had the highest echinocandin and fluconazole resistance levels, with those isolates that had double alterations being slightly more resistant to echinocandins but not as resistant to fluconazole. Although this analysis might be flawed due to the presence of compensatory *FKS* mutations or other alterations, such as stress response system changes that can also influence echinocandin resistance [28], this information is still important to build knowledge about the echinocandin resistance patterns of clinical isolates carrying different FKS amino acid substitutions.

Echinocandins include anidulafungin, caspofungin, micafungin, and the recently approved echinocandin rezafungin. Rezafungin is a long-lasting echinocandin that can be administered once weekly [29]. The PK/PD profile of rezafungin and high front-loaded exposures allow for higher concentrations to be achieved; some isolates carrying FKS mutations could be treated when tested in a neutropenic murine mouse model [30]. In an analysis based on Monte Carlo simulations to define the dosing schemes for rezafungin vs *C albicans* and *C glabrata*, all *C glabrata* isolates with an MIC value up to 4 mg/L would be treatable with the rezafungin-approved dosing of 400 mg per week, followed by 200 mg for 5 weeks [31]. The same authors discuss in a later publication that the dosing for other echinocandins has not been optimized as rigorously as the dosing for rezafungin [32, 33], indicating that if those evaluations were performed, the activity of the other echinocandins could be improved.

It was thought that the presence of FKS mutations was a predictor of clinical failure; however, recent data show that echinocandins can be treated in animal models with an optimized dosing schedule. These comprehensive analyses were used to justify a CLSI breakpoint 2 dilutions higher than the FDA-approved breakpoints for *C glabrata*. By using a breakpoint based on these optimized PK/PD parameters, 30% to 80% of the isolates carrying an FKS alteration in this study were susceptible to rezafungin. Other echinocandins, such as caspofungin and micafungin, displayed susceptibility rates as high as 45% for isolates harboring some of the FKS HS alterations.

It is important to note that fluconazole resistance among echinocandin non–wild type isolates was significantly higher when compared with the overall *C glabrata* population. This is a threat to patients since amphotericin B might be one of the only clinical available options for their treatment, highlighting the importance of development of new antifungal agents and/or strategies for the optimal use of available agents such as echinocandins [34]. The data on rezafungin suggest that in addition to the safety profile of the echinocandins and the once-weekly dosing that is convenient for outpatient management [35], this agent has been optimized in terms of PK/PD parameters to treat isolates with higher MIC results and could be an important option for infections caused by these isolates, including some isolates of *C glabrata* non–wild type to other echinocandins.

## Notes

***Acknowledgments*.** We acknowledge the medical centers participating in the SENTRY Antimicrobial Surveillance Program and the excellent technical assistance from Abigail Klauer, Paul R. Rhomberg, Danielle Beekman, and Abigail Scullin, who contributed to the testing for this study or editorial review of the manuscript.

***Author contributions*.** M. C.: conceptualization, funding acquisition, investigation, methodology, project administration, resources, supervision, formal analysis, methodology, validation, writing–original draft. L. M. D. and J. H. K.: data curation, formal analysis, investigation, methodology, validation, writing–review and editing. M. W.: conceptualization, funding acquisition, investigation, methodology, project administration, supervision, writing–review and editing.

***Data availability*.** The data that support the findings of this study are available on request from the corresponding author. The data are not publicly available due to privacy or ethical restrictions.

***Disclaimer.*** Melinta Therapeutics was involved in the design and decision to present these results, and JMI Laboratories received compensation for services related to preparing the manuscript. Pfizer was not involved in the collection, analysis, or interpretation of data.

***Patient consent statement.*** This study does not include factors necessitating patient consent.

***Financial support*.** This work was supported by Melinta Therapeutics.

## References

[1] Fidel PL Jr, Vazquez JA, Sobel JD. *Candida glabrata*: review of epidemiology, pathogenesis, and clinical disease with comparison to *C albicans*. Clin Microbiol Rev 1999; 12:80–96. 9880475 10.1128/cmr.12.1.80 PMC88907

[2] Pappas PG, Lionakis MS, Arendrup MC, Ostrosky-Zeichner L, Kullberg BJ. Invasive candidiasis. Nat Rev Dis Primers 2018; 4:18026. 29749387 10.1038/nrdp.2018.26

[3] Borman AM, Johnson EM. Name changes for fungi of medical importance, 2018 to 2019. J Clin Microbiol 2021; 59:e01811-20. 33028600 10.1128/JCM.01811-20 PMC8111128

[4] Pfaller MA, Diekema DJ, Turnidge JD, Castanheira M, Jones RN. Twenty years of the SENTRY Antifungal Surveillance Program: results for *Candida* species from 1997–2016. Open Forum Infect Dis 2019; 6(suppl 1):S79–94. 30895218 10.1093/ofid/ofy358 PMC6419901

[5] Rogers TR, Verweij PE, Castanheira M, et al Molecular mechanisms of acquired antifungal drug resistance in principal fungal pathogens and EUCAST guidance for their laboratory detection and clinical implications. J Antimicrob Chemother 2022; 77:2053–73. 35703391 10.1093/jac/dkac161 PMC9333407

[6] Castanheira M, Deshpande LM, Davis AP, Carvalhaes CG, Pfaller MA. Azole resistance in *Candida glabrata* clinical isolates from global surveillance is associated with efflux overexpression. J Glob Antimicrob Resist 2022; 29:371–7. 35577042 10.1016/j.jgar.2022.05.004

[7] Pappas PG, Kauffman CA, Andes DR, et al Clinical practice guideline for the management of candidiasis: 2016 update by the Infectious Diseases Society of America. Clin Infect Dis 2016; 62:e1–50. 26679628 10.1093/cid/civ933 PMC4725385

[8] Carvalhaes CG, Klauer AL, Rhomberg PR, Pfaller MA, Castanheira M. Evaluation of rezafungin provisional CLSI clinical breakpoints and epidemiological cutoff values tested against a worldwide collection of contemporaneous invasive fungal isolates (2019 to 2020). J Clin Microbiol 2022; 60:e0244921. 35249367 10.1128/jcm.02449-21 PMC9020363

[9] Alexander BD, Johnson MD, Pfeiffer CD, et al Increasing echinocandin resistance in *Candida glabrata*: clinical failure correlates with presence of FKS mutations and elevated minimum inhibitory concentrations. Clin Infect Dis 2013; 56:1724–32. 23487382 10.1093/cid/cit136 PMC3658363

[10] Smith HL, Bensman TJ, Mishra S, et al Regulatory considerations in the approval of rezafungin (REZZAYO) for the treatment of candidemia and invasive candidiasis in adults. J Infect Dis 2024; 230:505–3 38502709 10.1093/infdis/jiae146

[11] Food and Drug Administration . Antibacterial susceptibility test interpretive criteria. 2024. Available at: https://www.fda.gov/drugs/development-resources/antibacterial-susceptibility-test-interpretive-criteria. Accessed 19 August 2024.

[12] Locke JB, Pillar CM, Castanheira M, et al Outcomes by *Candida* spp in the ReSTORE phase 3 trial of rezafungin versus caspofungin for candidemia and/or invasive candidiasis. Antimicrob Agents Chemother 2024; 68:e0158423. 38526046 10.1128/aac.01584-23 PMC11064504

[13] Pfaller MA, Woosley LN, Messer SA, Jones RN, Castanheira M. Significance of molecular identification and antifungal susceptibility of clinically significant yeasts and moulds in a global antifungal surveillance programme. Mycopathologia 2012; 174:259–71. 22580756 10.1007/s11046-012-9551-x

[14] Clinical and Laboratory Standards Institute . M27 Ed4: reference method for broth dilution antifungal susceptibility testing of yeasts. Wayne, PA: Clinical and Laboratory Standards Institute, 2017.

[15] Clinical and Laboratory Standards Institute . M27 M44S Ed3: performance standards for antifungal susceptibility testing of yeasts. Wayne, PA: Clinical and Laboratory Standards Institute, 2022.

[16] Clinical and Laboratory Standards Institute . M57s Ed4: epidemiological cutoff values for antifungal susceptibility testing. Wayne, PA: Clinical and Laboratory Standards Institute, 2022.

[17] Castanheira M, Deshpande LM, Davis AP, Rhomberg PR, Pfaller MA. Monitoring antifungal resistance in a global collection of invasive yeasts and molds: application of CLSI epidemiological cutoff values and whole-genome sequencing analysis for detection of azole resistance in *Candida albicans*. Antimicrob Agents Chemother 2017; 61:e00906-17. 28784671 10.1128/AAC.00906-17 PMC5610521

[18] Healey KR, Perlin DS. Fungal resistance to echinocandins and the MDR phenomenon in *Candida glabrata*. J Fungi (Basel) 2018; 4:105. 30200517 10.3390/jof4030105 PMC6162769

[19] Centers for Disease Control and Prevention . Antimicrobial-resistant invasive candidiasis. 2024. Available at: https://www.cdc.gov/candidiasis/antimicrobial-resistance/?CDC_AAref_Val=https://www.cdc.gov/fungal/diseases/candidiasis/antifungal-resistant.html. Accessed July 2024.

[20] Perlin DS . Echinocandin resistance in *Candida*. Clin Infect Dis 2015; 61(suppl 6):S612–7. 26567278 10.1093/cid/civ791 PMC4643482

[21] Arendrup MC, Perlin DS. Echinocandin resistance: an emerging clinical problem? Curr Opin Infect Dis 2014; 27:484–92. 25304391 10.1097/QCO.0000000000000111 PMC4221099

[22] Pfaller MA, Messer SA, Rhomberg PR, Borroto-Esoda K, Castanheira M. Differential activity of the oral glucan synthase inhibitor SCY-078 against wild-type and echinocandin-resistant strains of *Candida* species. Antimicrob Agents Chemother 2017; 61:e00161-17. 28533234 10.1128/AAC.00161-17 PMC5527608

[23] Hoenigl M, Sprute R, Egger M, et al The antifungal pipeline: fosmanogepix, ibrexafungerp, olorofim, opelconazole, and rezafungin. Drugs 2021; 81:1703–29. 34626339 10.1007/s40265-021-01611-0 PMC8501344

[24] Ghannoum M, Arendrup MC, Chaturvedi VP, et al Ibrexafungerp: a novel oral triterpenoid antifungal in development for the treatment of *Candida auris* infections. Antibiotics (Basel) 2020; 9:539. 32854252 10.3390/antibiotics9090539 PMC7559578

[25] Hull CM, Parker JE, Bader O, et al Facultative sterol uptake in an ergosterol-deficient clinical isolate of *Candida glabrata* harboring a missense mutation in ERG11 and exhibiting cross-resistance to azoles and amphotericin B. Antimicrob Agents Chemother 2012; 56:4223–32. 22615281 10.1128/AAC.06253-11 PMC3421581

[26] Pham CD, Iqbal N, Bolden CB, et al Role of FKS mutations in *Candida glabrata*: MIC values, echinocandin resistance, and multidrug resistance. Antimicrob Agents Chemother 2014; 58:4690–6. 24890592 10.1128/AAC.03255-14 PMC4136002

[27] Desnos-Ollivier M, Bretagne S, Lortholary O, et al Echinocandins susceptibility patterns of 2,787 yeast isolates: importance of the thresholds for the detection of FKS mutations. Antimicrob Agents Chemother 2022; 66:e0172521. 35412354 10.1128/aac.01725-21 PMC9116480

[28] Garcia-Rubio R, Jimenez-Ortigosa C, DeGregorio L, Quinteros C, Shor E, Perlin DS. Multifactorial role of mitochondria in echinocandin tolerance revealed by transcriptome analysis of drug-tolerant cells. mBio 2021; 12:e0195921. 34372698 10.1128/mBio.01959-21 PMC8406274

[29] Thompson GR, Soriano A, Skoutelis A, et al Rezafungin versus caspofungin in a phase 2, randomized, double-blind study for the treatment of candidemia and invasive candidiasis: the STRIVE trial. Clin Infect Dis 2021; 73:e3647–55. 32955088 10.1093/cid/ciaa1380 PMC8662762

[30] Lepak AJ, Zhao M, VanScoy B, Ambrose PG, Andes DR. Pharmacodynamics of a long-acting echinocandin, CD101, in a neutropenic invasive-candidiasis murine model using an extended-interval dosing design. Antimicrob Agents Chemother 2018; 62:e02154-17. 29203480 10.1128/AAC.02154-17 PMC5786781

[31] Bader JC, Lakota EA, Flanagan S, et al Overcoming the resistance hurdle: pharmacokinetic-pharmacodynamic target attainment analyses for rezafungin (CD101) against *Candida albicans* and *Candida glabrata*. Antimicrob Agents Chemother 2018; 62:e02614-17. 29555634 10.1128/AAC.02614-17 PMC5971579

[32] Bader JC, Bhavnani SM, Andes DR, Ambrose PG. We can do better: a fresh look at echinocandin dosing. J Antimicrob Chemother 2018; 73(suppl 1):i44–50. 29304211 10.1093/jac/dkx448

[33] Zhao Y, Perez WB, Jimenez-Ortigosa C, et al CD101: a novel long-acting echinocandin. Cell Microbiol 2016; 18:1308–16. 27354115 10.1111/cmi.12640 PMC5096055

[34] Andes D, Diekema DJ, Pfaller MA, Bohrmuller J, Marchillo K, Lepak A. In vivo comparison of the pharmacodynamic targets for echinocandin drugs against *Candida* species. Antimicrob Agents Chemother 2010; 54:2497–506. 20385855 10.1128/AAC.01584-09 PMC2876357

[35] Sharma D, Vazquez JA. An evaluation of rezafungin: the latest treatment option for adults with candidemia and invasive candidiasis. Expert Opin Pharmacother 2024; 25:339–47. 38497379 10.1080/14656566.2024.2331775

